# Multifunctional applications of bamboo crop beyond environmental management: an Indian prospective

**DOI:** 10.1080/21655979.2022.2056689

**Published:** 2022-03-25

**Authors:** Rashmi Rathour, Hemant Kumar, Komal Prasad, Prathmesh Anerao, Manish Kumar, Atya Kapley, Ashok Pandey, Mukesh Kumar Awasthi, Lal Singh

**Affiliations:** a CSIR-National Environmental Engineering Research Institute (CSIR-NEERI), Nagpur, India; b Centre for Innovation and Translational Research, CSIR-Indian Institute of Toxicology Research, India; c Sustainability Cluster, School of Engineering, University of Petroleum and Energy Studies, Dehradun, India; d Centre for Energy and Environmental Sustainability, Lucknow, India; e College of Natural Resources and Environment, Northwest A&F University, Yangling, PR China

**Keywords:** Bamboo, bioenergy, land restoration, bamboo biochar, environmental benefits

## Abstract

Increasing population, industrialization, and economic growth cause several adverse impacts on the existing environment and living being. Therefore, rising pollutants load and their mitigation strategies, as well as achieving energy requirements while reducing reliance on fossil fuels are the key areas, which needs significant consideration for sustainable environment. Since India has considerable biomass resources, bioenergy is a significant part of the country’s energy policy. However, the selection of feedstock is a crucial step in bioenergy production that could produce raw material without compromising food reserve along with the sustainable environment. Higher growth capacity of bamboo species makes them a suitable lignocellulosic substrate for the production of high-value greener products such as fuels, chemicals, and biomaterials as well as an appropriate candidate for eco-restoration of degraded land. In that context, the current review discusses the multidimensional applications of bamboo species in India. The bioenergy potency of bamboo and probability of aligning its production, cultivation, and operation with economic and social development agendas are also addressed, making it an exceptional crop in India. Additionally, its fast growth, perennial root systems, and capability to restore degraded land make it an essential part of ecological restoration. Furthermore, this review explores additional benefits of bamboo plantation on the environment, economy, and society along with future research prospects.

## 1. Introduction

India is the third-largest energy consumer in the world and the fourth-largest consumer of petroleum and crude oil, spending 45% of its export earnings to import energy [1]. The energy demand has risen substantially, corresponding with its economic development, urbanization, and population growth. In India, fossil fuels including coal, oil, and natural gases are the primary sources of energy, signifying the country’s major share of the energy mix [2]. The Indian Government has vowed to decrease greenhouse gas (GHG) emissions in decarbonizing its economy. India focused on reducing GHG emissions by 33–35% by 2030 in its Nationally Determined Contribution (NDC) to the United Nations Framework Convention on Climate Change (UNFCCC) in 2015. As the main goal of the UNFCCC is to stabilize GHG concentrations in the atmosphere at a level that prevents harmful interference with the climate system [3]. On the other hand, India remains confronted with obstacles in the energy market. Despite improvements, the national energy security remains below standard and unsatisfactory compared to several other developed and emerging nations. Today, several remote regions lack access to advanced energy sources, relying on the direct combustion of conventional sources of bioenergy like fuelwood, which can cause health issues and have adverse effects on the environment.

The foundation of sustainable development is ensuring equitable access to affordable, secure, and renewable energy services [4]. Several other objectives are inextricably related to it, including climate action, healthy living, no hunger, and reducing poverty. The Indian Government is also dedicated to delivering energy to its rising inhabitants for the economic growth and the upliftment of 9% of the inhabitants living in extreme poverty via the National Energy Policy [5]. Energy diversification, environmental protection, and domestic energy resources are all emphasized in the policy. India aims to upsurge the share of renewable energy in its energy mix as the global community explores clean and efficient renewable energy to achieve the United Nations Sustainable Development Goals (UN-SDGs) [6]. Presently, renewable energy accounts for 9.8% of the national energy mix, with the nation targeting to raise its total final consumption to 13% by 2030 and 17.5% by 2040. Bioenergy is projected to contribute the most to this proposed renewable energy mix, accounting for 21.2% [5]. Bioenergy is a substantial renewable energy source extracted from lignocellulosic biomass/plant residue [7,8], algal biomass [9,10], organic fraction of municipal solid waste (OFMSW) [11,12], municipal sewage sludge [13–15], plastic waste [16] to produce electricity, heat, or liquid transportation fuels. It comprises gases (such as biogas, producer gas, syn-gas biohydrogen, etc.), liquid biomass (such as bioethanol, biodiesel, bio-oil, butanol, etc.), and solid biomass (such as briquettes, charcoal, firewood, pallets, wood chips, etc.) derived from agricultural waste, forests, plants, and other sources [17–19].

India has immense land resources with significant climate, elevation, soil, and physiographic variations, allowing for the development of bioenergy from various plants [20,21]. Though expanding bioenergy plantations can conflict for water and land, it poses a threat to biodiversity conservation and food production [22,23]. In India, bioenergy is primarily derived from oil palm, a controversial crop that is highly disputed. It has been discovered that it causes extensive deforestation, peatland runoff, and other social and ecological aspects. Thus, it is essential to identify suitable crops for bioenergy feedstock to minimize the undesirable effects on biodiversity, food security and maximize bioenergy production [24–26].

**Figure 1.**
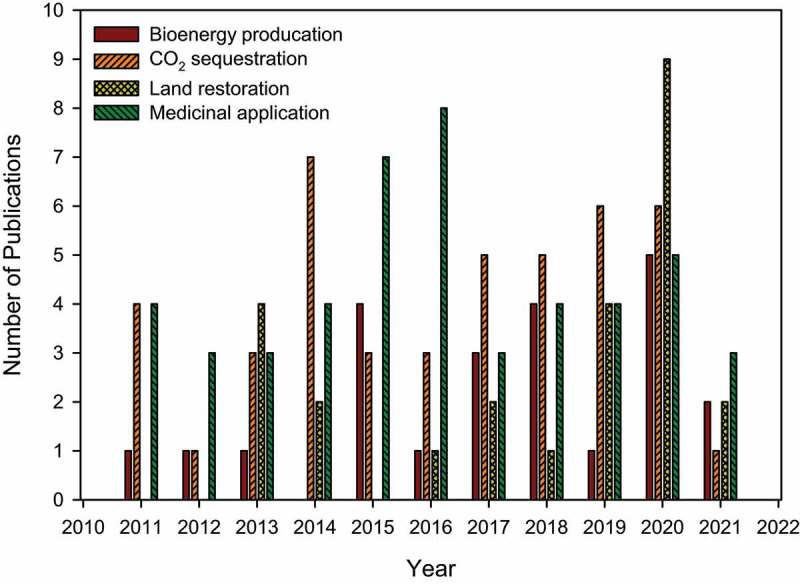
The number of publications on bioenergy production, CO_2_ sequestration, land restoration, and medicinal application of bamboo (Source: Web of science).

Bamboo is part of the *Poaceae* grass family and the subfamily of *Bambusoideae*, which grows in many parts of the globe [27]. It has more than 1575 species from the 111 genera, which are very distinct from the common grasses owing to the width, size, height, and branches on the internodes [28]. In terms of the whole bamboo community, India is the second-largest cultivars of bamboo species, with 160 species, after China, which has 800 species. Bamboo is best known for its three distinct features: flowering, rapid growth, and increased physical and mechanical characteristics [29]. Bamboo has a remarkable ability to store and capture carbon (C) and a high nitrogen (N_2_) fixation ability, making it very appealing to biologists [30]. Humans have used bamboo for thousands of years, and it is used in India as a fiber, firewood, and food source. The sturdy and versatile woody stems are often referred to as ‘timber of the poor’ because they are used as a building material [31]. In recent years, bamboo has been used for several purposes other than those traditionally associated with it because of advances in technology and growing demand for environmentally friendly products and services (Table 1). For instance, it is used to produce and design long-lasting equipment, furniture, and construction materials. It has over 1500 implementations and can be used in many different ways [32]. Bamboo is being investigated as a potent raw material to produce electricity via power stations and biofuels to replace renewable resources owing to its higher efficiency, fuel characteristics, and a short rotation [33]. Bamboo has been utilized in India for decades but is still a developing biomass feedstock. Furthermore, several published studies on the use of bamboo in bioenergy production, CO_2_ sequestration, land restoration, and medicinal applications have emerged in the last decade, as shown in Figure 1.

**Table 1.** Selected studies related to bamboo plantation and their key highlights

Year	Country	Highlights of study	Reference
2020	India (North Eastern Region) and Bangladesh	NER can grow the bamboo sector by capitalizing on the availability of natural bamboo resources; Bangladesh has a booming furniture sector, notably in wood, and may buy semi-processed and finished bamboo products from NER as well as invest in the region; NER can serve as an upstream provider of semi-processed bamboo to enterprises in other clusters and areas.	[44]
2020	India (marginal ecologies; saline and drought lands of Surendranagar, Bhavnagar and Aravali districts of the Gujarat state)	Energy crops for biofuel production are acceptable, but land availability is a priority; Sorghum, pearl millet, and bamboo were researched as energy crops because of their high biomass; Bamboo with a high biomass exhibited the greatest economic, energy, and environmental benefits; Integrated policy assistance may be used to implement bamboo plantation in a multicrop system.	[42]
2020	India	Provides information on twenty-seven commercial bamboo species discovered during field research in India; Clustering algorithms were used to determine the market worth of each of the twenty-seven species; Creation of relevant intervention plans in various industrial sectors in terms of marketing and technological advancement	[28]

In that context, the present review comprehensively spotted the light on, multidimensional application of bamboo, with a major emphasis on bamboo as an energy crop in India. Furthermore, this review examined the land restoration and environmental management potential of bamboo plantations critically. Finally, the economics of bamboo plantations in India and their opportunities and challenges have been discussed in detail and prospects for future research.

## 2. Bamboo for bioenergy production and environmental management

After China, India is the world’s second-richest nation in bamboo genetic wealth [34]. India is home to around a quarter of the world’s bamboo plants, present in almost every province. In India, bamboo is commonly cultivated in the Western Ghats and Northeast India. The annual production of bamboo is approximately 4.6 million tons (Mt), ranging from 0.2 to 0.4 t ha^−1^, with an average of 0.33 t. Bamboo accounts for 13% of the country’s overall forest sector. The country’s total bamboo-bearing region is projected to be 160,037 km^2^. According to the India State of Forest Report (ISFR) 2017, the region for bamboo cultivation has increased by 3229 km^2^ [35]. As per the Forest Survey of India (FSI) report, Maharashtra has a bamboo-bearing land of 11,465 km^2^, while Vidarbha region producing more than 90% of the total bamboo biomass [36]. The global bamboo industry is expected to be worth 12,000 million USD, with India accounting for just 4.5% of that despite holding 31.1% of the world’s overall bamboo growing region [37]. The gross carbon reserve in forests is projected to be 7124.6 Mt in the current evaluation. Compared to the previous estimate in 2017, the country’s carbon stock has increased by 42.6 Mt [35]. It also gives around 40 t of biomass ha^−1^, which projected its potentiality towards the generation of bioenergy and environmental management [30,38].

### 2.1. Energy production and fuel characteristics

In the world’s developed nations, woody biomass is the primary source of electricity. According to the Food and Agriculture Organization (FAO), woody biomass provides around 13% of the world’s primary energy [39]. In the tea-producing state of Assam, the longest grass tribe, also referred to as green petrol, produces 60 million liters of ethanol per year. That would be enough to satisfy the northeastern region’s required mixing needs for gasoline [40]. Woody bamboos grow quickly and can generate up to 50 Mt of biomass acre^−1^ y^−1^, that could convert into a range of energy products such as charcoal, biogas, and biofuels [38,41]. Feedstock for biofuel development is a major struggle for any nation, as biofuel crops conflict with grain, fodder, and fiber for scarce land resources [42]. Bamboo species have an estimated annual biomass growth of 22–44 Mt ha^−1^ y^−1^, which is far higher than pine (25–30 Mt ha^−1^ y^−1^) and equivalent to eucalyptus (30–40 MT ha^−1^ y^−1^) forests [38]. Bamboo contained favorable fuel properties, including a low ash content and low alkali index, as well as a lower heating value, which is greater than other biomass feedstock, grasses, and straw but lower than many woody biomasses [28,43]. Pyrolysis had been proposed as a viable thermochemical process for producing biochar, bio-oil, and syngas from biomass. Bamboo was chosen as the pyrolysis feedstock for producing pyrolytic products due to its capacity to grow sustainably with high density and global abundance [44]. Bamboo also has excellent fuel qualities, like high caloric values and volatile contents, as well as lower moisture and ash content, making it an excellent choice for biofuel generation. Bamboo often has a strong lignocellulose content as compared to many other biomass species. These characteristics may vary depending on plants, region, maturity level, and management activities etc. Sadiku et al. [39] found that the chemical composition of *Bambusa vulgaris* varied from 4 to 7% for extractives, 39 to 46% lignin, and 61 to 78% cellulose with a gross calorific value ranging from 1810.90 cal kg^−1^ to 4160.60 cal kg^−1^, with an average of 3157.80 aged between 2 and 4 years. Kumar and Chandrashekar [41]. Evaluated the fuel attributes of five key bamboo species (*Bambusa balcooa, Bambusa bambos, Dendrocalamus brandisii, Dendrocalamus stocksii* and *Dendrocalamus strictus*) and concluded that species differed in their combustion behaviour, owing to changes in physical and chemical parameters. Nevertheless, its total heating value and structure are about that of herbaceous wood and hardwoods. Bamboo has comparable fuel properties to other biomass feedstocks [28,42,44]. In thermoelectric plants and the building industry, the bamboo feedstock is used on an industrial scale by direct combustion or cogeneration of heat and electricity [38]. The fuel characteristics of common bamboo species found in India were shown in Table 2.

**Table 2.** Fuel characteristics of common bamboo species in India

Bamboo species	Ash (%)	M (%)	VM (%)	HV (Kcal Kg^−1^)	FC (%)	CEL (%)	HCEL (%)	LIG (%)	C (%)	H (%)	Reference
*Bambusa balcooa*	0.84	7.80	81.36	4349.30	10.00	58.10	23.70	25.0	45.58	6.31	[129,130]
*Bambusa bambos*	14.00	9.80	71.40	4507.00	4.80	NA	NA	NA	44.87	7.60	[130]
*Bambusa tulda*	4.50	15.00	80.30	4394.00	15.20	NA	NA	NA	43.13	2.97	[131]
*Bambusa vulgaris*	1.85	9.64	69.92	4359.00	2.81	27.00	30.60	12.7	43.78	6.65	[38,130]
*Dendrocalamus asper*	2.70	5.80	71.70	4198.00	5.19	40.70	NA	27.1	43.06	6.57	[129,130]
*Dendrocalamus giganteus*	2.41	9.60	68.48	4499.00	5.70	24.45	40.47	16.68	NA	NA	[38,130]
*Dendrocalamus strictus*	11.10	8.82	75.08	4294.00	5.01	NA	NA	NA	46.87	6.19	[130]
*Phyllostachys edulis* (tortoise shell bamboo)	1.44	9.00	73.81	4225.10	4.58	38.10	23.10	25.4	44.25	6.16	[130,131]
Bamboo leaves	12.30	7.70	68.70	3756.00	11.30	47.00	21.00	NA	NA	NA	[132]
*Phyllostachys edulis* (Moso Bamboo)	1.750	10.83	75.48	4612.00	12.26	46.46	24.47	26.84	47.34	5.83	[131]

M: Moisture; VM: Volatile matter; HV: Heating Value; FC: Fixed carbon content; CEL: Cellulose; HCEL: Hemicellulose; LIG: Lignin; C: Carbon; H: Hydrogen; NA: Not Available

### 2.2. Carbon sequestration by bamboo

One of the most severe challenges of the new millennium is global warming. The Kyoto Protocol emphasizes the scientific community’s deep concern about rising carbon emissions caused by development activities [45]. Carbon sequestration is a recent approach to climate change mitigation strategies that have received considerable attention. Carbon sequestration from the atmosphere by plants is a highly efficient, natural, and low-cost technology with enormous carbon collecting and storage potential [46]. As one of the most productive and fastest-growing plants on the planet, Bamboo has the potential to be a valuable carbon sink due to its decay-resistant litter. The sequestration capacity of bamboo is determined by its growth and life cycle, varying from species to species. Every year, one hectare of bamboo consumes approximately 17 t of carbon. Bamboo stands cover 36 million hectares globally, accounting for 3.2% of the total forest area [47]. In Asia, India is the leading producer of bamboo (nearly 11.4 million hectares), accounting for approximately half of the total area reported for Asia. The dry matter weight in common thorny bamboo, *Bambusa bamboos*, has been recorded to be 122, 225 and 286 t ha^−1^, respectively, at the ages of 4, 6, and 8, and is comparable to the 10-year-old quick-growing *Casuarina equisetifolia* or *Eucalyptus tereticornis* plantation. When contrasted to *Tectona grandis, Dalbergia sissoo*, and *Acacia nilotica* at ten years, the *Dendrocalamus strictus* accumulates a lot of biomasses per hectare in three years [35].

India must promote bamboo cultivation in order to enhance forest cover and capture rising CO_2_ levels. As part of the goals, it set for itself under the Paris Agreement in 2015, India told the UNFCC that afforestation would provide an additional carbon sink of 2.5–3 GtCO2e (gigatonnes of carbon dioxide equivalent) by 2030 [48]. Research was conducted in the Barak valley in the northeastern states of India, involving three species *Bambusa Balcooa* (26%), *Bambusa Cacharensis* (46%), and *Bambusa Vulgaris* (28%). The bamboo plantation has a total carbon stock of around 120.75 t ha^−1^. The storage of carbon in the aboveground biomass, soil (up to 30 cm depth), and litter floor mass was 61.05 t ha^−1^, 57.3 t ha^−1^ and 2.40 t ha^−1^, respectively [49]. Another research was conducted on *Phyllostachys pubescens*, the most common Japanese bamboo species. The total carbon output was predicted to be 32.81 t ha^−1^ y^−1^, with aboveground biomass accounting for 18.11 t ha^−1^ in and belowground biomass accounting for 11 t ha^−1^. Carbon storage by *Dendrocalamus strictus* plantations in Indian dry deciduous woods was calculated to be 96.35 t ha^−1^. Below ground accounted for 26% of the total, while above ground accounted for 74%. The annual carbon sequestration rate of 9.89 t ha^−1^ was calculated in Taiwanese Makino Bamboo using the premise of selective harvesting of 20% clumps each year. Makino Bamboo was determined to have the carbon storage value of a 29-year-old Taiwanese red cypress in just 8.3 years and a 33-year-old Japanese cedar in 14.8 years [50]. When Moso Bamboo was substantially maintained, the annual carbon sequestration rate was reported to be 8.1 t ha^−1^ y^−1^. It was shown to increase up to 12.7 t ha^−1^ y^−1^ (fertilizing annually, tending, harvesting bamboo and bamboo shoots regularly) with careful management. It sequesters carbon at a rate that is 2–4 times that of tropical rainforests and pine plantations and 3.6 times that of Chinese Fir. Annual carbon sequestration by *Bambusa bamboos* and *Schizostachyum pergracile* was determined to be 20.5 t ha^−1^ y^−1^ and 26.96 t ha^−1^ y^−1,^ respectively [51].

It was observed that different bamboo species could achieve carbon storage and sequestration rates of 30–145 t ha^−1^ and 1.3–24 t ha^−1^ y^−1^, respectively [50]. The global average carbon storage would be approximately 86 t ha^−1^ in forest vegetation, which can effectively maintain bamboo plantation could easily exceed. Bamboo must be appropriately managed to achieve maximum CO_2_ sequestration [52]. This is important to note that bamboo management entails the harvesting of specific columns regularly. The amount of carbon stored in harvested culms is also calculated and compared to other forest-based options.

### 2.3. Restoration of degraded land

Land degradation refers to a permanent or temporary reduction in the productive potential of land, which has severe implications for the environment, biodiversity, and agriculture [53]. This may also exacerbate poverty in developing nations because it impacts people relying on land-based economic activity [54]. As a result, UN-SDGs and conventions such as UNFCCC, the United Nations Convention to Combat Desertification (UNCCD), and the Convention on Biological Diversity (CBD) have prioritized land restoration in addressing the land degradation crisis. Soil erosion caused by water, wind, and degradation of vegetation cover has been the leading causes of land degradation in India [55]. According to the Desertification and Land Degradation Atlas of India, created by the Space Applications Centre for the duration 2011–2013, 96.4 million hectares, or 29.32% of the countrys total geographic area, are decertified or deteriorated [56]. India has taken several steps to address this issue, including restoring degraded areas.

However, restoration attempts are frequently hampered by the biophysical and legal constraints of land and the high expenses of reclamation. Biophysical techniques will be required to restore extensive degraded lands in India, necessitating enormous time and financial investments [57]. The restoration cost is difficult to assess because it depends on various factors, including the location, methods, and level of degradation. However, according to the literature, the costs of employing forest species to restore a hectare can easily reach USD 2300 [58]. This implies that productivity and financial returns will be slow, making investors and farmers hesitant to invest in restoration. One common cost-cutting strategy in such cases is to plant fast-growing species that can thrive in low-fertility soils with minimal management intervention [40]. As a result, bamboo is a unique plant for land restoration due to its benefits, economic reserves, and ecological properties.

Compared to other conventional biomass sources, bamboo grows well in marginal and degraded soils with low fertility and requires little water or fertilizer [42]. This means that bamboo, even with fewer resources, can thrive in severely degraded areas where other native species cannot grow. Furthermore, the leafy mulch, dense foliage, extensive fibrous rhizome, and root bamboo systems prevent soil erosion, retain water and stabilize the soil [59,60]. Bamboo leaf litter enriches the soil with organic matter and improves the fertility of degraded soil. Furthermore, bamboo does not necessitate significant investments and, once installed, can be operated without the need for extensive maintenance. Bamboo proliferates and can be harvested consistently without replanting for 3–4 years, resulting in higher returns on capital, attracting farmers and investors [61]. As managed bamboo becomes more widely available, households will substitute bamboo slats for firewood, a renewable alternative; bamboo will thus aid in reducing deforestation. Aside from that, bamboo contributes to landscape diversity by providing habitat and food for various animals, birds, and insects.

As part of the Bonn Challenge, several International Network for Bamboo and Rattan (INBAR) member countries promote bamboo for land restoration. India has made significant commitments to restoration, recognizing the significance of restoration to the country’s socioeconomic well-being and the achievement of the US-SDGs. For example, a bamboo-based landscape proposal that began in 1997 has successfully rehabilitated over 85,000 hectares of degraded land, even supporting thousands of livelihoods [58]. In 2015, it pledged to rehabilitate 13 million hectares of damaged and deforested land by 2020 and a further 8 million hectares by 2030 as part of the Bonn Challenge [62]. Among the current bamboo-based restoration programs are the Philippines’ plan to recover at least 500,000 hectares and the Chinese State Forest Administrations plan to regain three million hectares [63]. Therefore, Bamboo can play a crucial role in reclaiming degraded land and alleviating poverty in rural areas.

## 3. Value-added products from bamboo

Bamboo can be a potential source of social, economic, and environmental development because of its unique properties, including a faster growth rate, less management, a limited amount of growth requirements, and less expertise [63,64]. This plays a significant role in uplifting rural low-income communities [65]. Natural bamboo forests cover approximately 10.03 million hectares in India, with two-thirds of that area located in the countrys northeast. Only 35% of this land is used by the rural for various purposes such as housing and pulp. The waste biomass of bamboo shoots after harvesting accounts for approximately 70% of its substantial quantities. The solid waste residues of the sheath and basal section are also used for various purposes, such as pulp production during the processing of by-products. Bamboo shoot tips and young shoots are edible because of their high nutrient content and health benefits from bioactive compounds. Bamboo can be used to produce a variety of value-added products [50,66]. Table 3 demonstrated the significance of different bamboo species in multi-utility implementations.

**Table 3.** Significance of different bamboo species for multi-utility implementations

Value-Added Products	Bamboo Species	Activities/Properties	Reference
Medicinal Application	*Bambusa arundinacea*	Anti-inflammatory, antiulcer activity	[67]
	*Bambusa bambos*	Estrogenic effects	[133]
	*Bambusa Vulgaris*	Anti-oxidant, young shoots used to treat hepatitis and measles	[68]
	*Bambusa balcooa*	Anti-hyperglycemic	[134]
	*Bambusa tulda*	Anti-oxidant	[135]
	*Bambusa blumeana*	Antiulcer	[69]
	*Dendrocalamus asper*	Anti-oxidant	[68]
	*Dendrocalamus latiflorus*	Anti-diabetic	[136]
	*Phyllostachys nigra*	COVID19 inhibitory, anti-cancer, anti-inflammation, cardio-protective	[69]
	*Phyllostachys edulis*	Antitumor activity, lipid lowering	[68]
	*Phyllostachys heterocycle*	Anti-cancer activity	[70]
	*Sasa senanensi*	Anti-cancer, anti UV, anti-HIV	[70]
Bamboo Biochar			
	*Dendrocalamus giganteus*	lithium-sulfur (Li-S) batteries	[137]
	*Dendrocalamus latiflorus munro*	rich in inorganic nutrients	[88]
	*Moso bamboo*	Bio-oil production	[138]
	*Phyllostachys praecox*	Effects on eco enzymatic stoichiometry	[89]
	Unspecified bamboo species	Arsenic contaminated water treatment, enhancing soil water holding capacity, in geochemical speciation and up- taking of heavy metals	[139–141]
Composite Materials	*Bambusa balcooa*,	Handicraft items	[102]
	*Bambusa nutans*	Biodegradable products preparation	[98]
	*Dendrocalamus asper*	Used in low-cost polymer	[142]
	*Dendrocalamus strictus*	Strong and elegant used in construction works	[50]
	*Guadua angustifolia*	Adhesive property used in furniture	[109]
	*Phyllostachys edulis*	Reinforcement provided by fibers	[50]
Food Source	*Bambusa arundinacea*	Regulates levels of vitamins	[103]
	*Bambusa balcooa*	Rich in fibers and protein	[105]
	*Bambusa tulda*	Use as fat cutter	[106]
	*Bambusa polymorpha*	Regulates levels of vitamins	[103]
	*Bambusa vulgaris*	Rich in minerals and dietary fibers	[106]
	*Dendrocalamus asper*	Rich in proteins and carbohydrates	[105]
	*Dendrocalamus gigantea*	Rich in minerals and dietary fibers	[106]
	*Dendrocalamus hamiltonii*	Rich in minerals	[104]
	*Gigantochloa manggong*	Rich in minerals and dietary fibers	[106]
	*Melocanna baccifera*	Rich in vitamins	[107]
	*Phyllostachys edulis*	Artificial sweetener	[112]
	*Phyllostachys pubesens*	rich in carbohydrates and vitamins	[67]
	*Yushania alpina*	Used in prebiotic	[107]

### 3.1. Medicinal potency

Bamboo has shown enormous potential for medicinal properties since ancient times. Bamboo salt, vinegar, and extracts have been used in pharmaceutical preparation to control and regulate diabetes, cholesterol, and hypertension levels. *Bambusa arundinacea* leaf extract has anti-inflammatory, antioxidant, antiulcer, and anthelmintic properties, which have led to its use in phytopharmaceuticals. The burned root and bark of bamboo are used to treat arthritis, bleeding gums, and skin eruptions, respectively [50,67]. The leaves and seeds of the bamboo plant are used to treat hemoptysis (anticoagulation) and urinary discharges. The pyrolyze extract of *Bambusa bambos* has antimicrobial and antifungal activity, which protects neurons from oxidative stress and aids digestion and appetite stimulation [67]. *Phyllostachys nigra* has been shown to have anti-inflammatory activity by inhibiting NF-kB-induced gene expression, which also helps reduce cholesterol and high-density lipoprotein and aids in weight loss. *Phyllostachys* sp. has antioxidant activity, whereas *Bambusa arundinacea* leaf extract has antithrombotic activity [68].

The current global outbreak of severe acute respiratory syndrome, SARS-CoV-2, also known as COVID-19, began in Wuhan (China) and has spread to nearly 187 countries. A study discovered that orientin, a natural flavonoid found in black bamboo with phenomenal properties such as anti-inflammation, cardioprotective, neuroprotective, anticarcinogenic, and antiviral, effectively inhibited SARS-CoV-2 [69]. The orientin molecule binds to the host receptor overlapping residues of GPR78, which is the binding region of the SARS-CoV-2 spike model, demonstrating that the orientin compound from black bamboo can interfere with the interaction of the COVID-19 virus.

Bamboo has anti-cancer properties, according to several studies. One study on cancer cell lines found that the extract of the bamboo shoots of *Phyllostachys heterocycla* and *Phyllostachys pubescens* causes apoptotic cell death in HepG2, MCF-7 cells and acts as an enzymatic inhibitor of tyrosine kinase [70]. Similarly, a study on bamboo salt conducted in Korea found that it has *in-vitro* anti-cancer properties against human colon cancer cells. Bamboo salt has anti-cancer and anti-inflammatory properties, which allow it to inhibit human colon cancer-causing cell HCT-116 by up to 53% and affect metastasis by assisting in the apoptosis of these cells [69]. A similar study was carried out on *Phyllostachys heterocycla* to understand its anti-inflammatory response. The study was conducted on rats, and the crude methanolic extract was used in various doses, resulting in an increase in the level of four inflammatory markers (NF-B, IL-1, TNF-, and IL-6) present in the serum and concluding that the *Phyllostachys heterocycla* extract possessed potent anti-inflammatory properties [70]. *Sasa coreana Nakai* (SCN), a Korean bamboo variety, has anti-inflammatory properties for the skin and ears. Khan et al. [71,72] observed morphological changes in mice treated with SCN leaf extract, implying that it can be used as a therapeutic agent against such inflammation.

Increased glucose levels in humans can lead to various problems, including decreased lifespan, glucose toxicity, and so on. A study was carried out using the *Caenorhabditis elegans* model system, and leaves extract of black bamboo *Phyllostachys nigra* were used on transgenic mutant worms carrying the DAF-16 transgene. The results show that incorporating bamboo leaf extract extends the worm lifespan and regulates high glucose levels, reducing glucose toxicity [73]. Yellow and green bamboo have been used as a food source and medicine in Indonesia since ancient times. They are widely used by the locals because they are readily available and inexpensive in comparison to other sources, and they contain a high concentration of essential compounds such as phenol, flavonoids, vitamin E, and so on, allowing them to be used as an antioxidant [74]. The therapeutic and medicinal properties of these bamboos demonstrated effective use as a medicinal plant.

### 3.2. Bamboo biochar

Biochar is a relatively stable carbonaceous substance synthesize using different feedstocks such as lignocellulosic waste [75,76], municipal waste [77,78], plastic waste [16] at elevated temperatures (> 400°C) in absence of oxygen supply. Owing to its excellent cation exchange capacity, porosity, high surface area, and surface functional groups, it has been extensively applied in removal of organic and inorganic contaminants [79,80] additives in anaerobic digestion and composting [11,81], and soil amendment [78]. Due to excessive availability of bamboo biomass, it can be considered as an excellent feedstock for production of biochar via thermochemical conversion routes [59]. Pyrolysis is primarily used to convert feedstock into carbon-rich products such as biochar in oxygen free environment. Bamboo contains nearly 50% carbon, making it suitable for use as biochar for industrial and agricultural purposes. Bamboo charcoal/char can replace wood charcoal due to its high calorific value (nearly half of the petroleum oil) [39,41]. Bamboo biomass is pyrolyzed in an oxygen-free environment to produce biochar and bio-oil, which can be further use to remove contaminants from diverse environmental matrices and also act as a potential energy resource [82]. Bamboo biochar has a high degree of stability, a well-developed pore structure, a wide surface area, a high capacity for cation exchanges, and many functional surface groups such as carbonyl, carboxylic, hydroxyl, quinone, and phenolic groups which make it potential carbonaceous material for energy and the environment [77,83]. The application of biochar can limit the release of greenhouse gases such as CH_4_ and N_2_O during the process of composting [84]. Gong et al. [83] studied the impact of bamboo biochar on the growth and reproduction of *Eisenia fetida*, finding that introducing 6% bamboo biochar enhanced earthworm biomass, the number of juveniles and cocoons, and the degradation of lignin substrate during vermicomposting by up to 13.98%. Zhang et al. [85] used bamboo biochar particle electrodes in a three-dimensional electrochemical reaction system to treat coking wastewater. They discovered that using Ti-Sn-Ce/BC particle electrodes, the dissolved organic carbon (DOC) and chemical oxygen demand (COD) removal rates of coking wastewater were 74.66% and 92.91%, respectively, at a current density of 30 mA/m^2^ and an electrolytic duration of 120 minutes. Additionally, the UV_254_ of coking wastewater decreased from 7.72 to 1.22 cm^−1^, indicating the biodegradability of the organic pollutants.

Bamboo biochar has a wide range of environmental applications that have emerged in the last decade. When biochar is mixed with animal feed, it acts as a better supplement to their diet and has positive effects such as improved growth, egg yield, pathogen resistance, etc. making it a potential additive to animal feed in animal farming [86]. The efficacy of varied concentrations of bamboo biochar on nutrient conservation was investigated using gaseous emissions from poultry manure composting [81]. The overall carbon and nitrogen losses were significantly reduced when biochar levels were increased from 542.8 to 148.9% and 53.5 to 12.6%, respectively. With a high concentration of biochar added to composting, enzyme activity related to C and N metabolism increased significantly. Simultaneously, changes in total Kjeldahl nitrogen (TN), total organic carbon (TOC), and maturity indices revealed a high-quality composting product.

**Figure 2.**
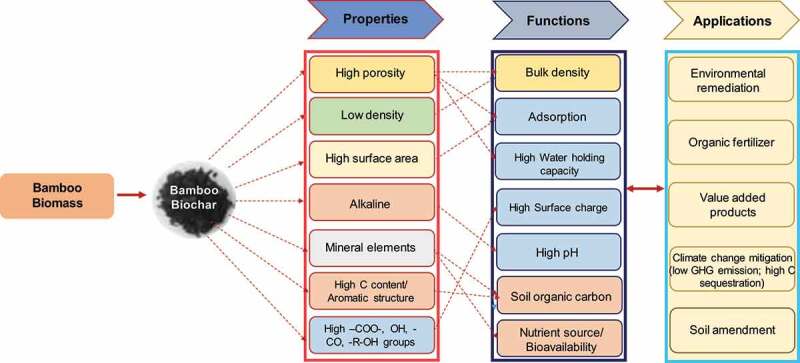
Framework for understanding the effects of bamboo biochar application on soil properties and climate change mitigation.

Bamboo biochar has been used as a soil supplement for crop production because it enhances soil fertility, decreases exchangeable aluminium levels, improves water retention, stimulates soil aggregate formation, reduces N and phosphate leaching, and increases microbial activity [83]. A study on *Solanum lycopersicum* using biochar pyrolyzed at different temperatures revealed a significant increase in plant growth with increased sugars (glucose and fructose), ascorbic acid content [87,88]. This demonstrates that the use of biochar as desirable amendments can impact plant growth and development. A study was conducted to produce bamboo biochar from the shoot of *Dendrocalamus latiflorus Munro* at operating temperatures ranging from 300°C to 500°C under N_2_ flow. When amended to soil, bamboo biochar exhibits a good value of inorganic nutrients, improved water holding capacity, and bulk density of soil, moisture retention, control soil pH, and immobilize toxic metal ion [88]. The role of bamboo biochar in soil fertility and climate change mitigation was shown in Figure 2. Similarly, a study was conducted to investigate the variation in soil enzyme activity and microbial C and N use, when *Phyllostachys praecox* was used to prepare biochar at three different pyrolysis temperatures (350, 500, and 700°C). The results revealed a significant increase in pH, total carbon (TC) and N concentrations in the biochar amended soil [89]. Furthermore, this study also showed an increase in enzyme activities, which gradually increases microbial nutrient metabolism. Increased in dehydrogenase activities in tainted soil has been observed by Demisie et al. [90] after soil amended with 0.5% (w/w) biochar prepared using bamboo biomass and oak wood as feedstock. Additionally, urease and β-glucosidase activities were also enhanced after amending bamboo and oak wood biochar in the soil. Although, mechanisms associated with biochar amendment in soil and improvement in enzymatic activities are still in early stages stage and needs extensive investigation.

### 3.3. Bamboo residue

Several investigations have demonstrated that bamboo residue can be utilize as a feedstock for generation of bioenergy [91,92]. Higher cellulosic (sugar) content of bamboo residue in comparison to other woody biomass make them a potential feedstock for production of diverse range of chemicals and fuels, such as lactic acid bioethanol, butanol etc [93]. Bamboo residue can also be applied in generation of biogas from anaerobic digestion. Furthermore, bamboo residue has significant inherent characteristics, such as high cellulosic and low lignin content which make it a suitable material for generation of liquid fuel/bioethanol [58,94]. It was estimated that 143 L of bioethanol can be produced from one ton of bamboo [95]. In contrast, for extracting 1 kg bioethanol from bamboo residue, 6.2 kg feedstock, 8.5 kg H_2_SO_4_, and 65.8 L of process water are needed [96]. In addition to this, owing to imperative characteristics of bamboo residue, such as lower ash content and alkali index, make them a potential feedstock for generation bioenergy in comparison to woody biomass [92]. Nevertheless, bamboo residues required pretreatments, which improve cellulosic and hemicellulosic content by removing lignin fraction. The process of pretreatment can also improve enzymatic saccharification for extraction of sugars [97]. In general, bamboo residue can be successfully applied as an important feedstock for generation of fuels and diverse range of chemicals.

### 3.4. Composite material

Bamboo emerged as the most peculiar material for construction in India, South America, Africa, and some parts of Asia because of its properties such as elasticity, wooden-like strength, elegance, and lightweight. It has been used primarily in earthquake-prone regions and also prevents fire as it contains a high amount of silica [50]. The composite material made from bamboo fiber is known as bamboo scrimber that increased the interest of manufacturers due to its excellent mechanical properties, which can be designed and shaped using technology such as heat treatment, adhesives, defibering, and so on, resulting in incredible results [98,99]. Bamboo has proven its usefulness as a healthy, productive composite material as a widely accessible, low-cost, and environmentally acceptable raw resource because its fibers serve as reinforcement, which is a low-cost polymeric, biodegradable, and long-lasting substance [100]. They are processed using various methods and applied in the development of high-quality composites.

For many years, timber grass has been popular as a building material. Because of its enormous potential, it has made significant contributions to the rural economy and the establishment of sustainable development. Because of their elasticity and tensile strength, they can be used as an alternative to steel in building materials [101]. They are frequently used in furniture because of their attractive designs, resistance to termite attack, and low cost [102]. Chairs, tables, sofas, dining tables, cots, school, and office furniture are all in high demand, even though bamboo is also used to make handicraft items such as baskets and lampshades, showpieces, decorative items, and some rural regular-household utility that could have significant long-term benefits for the rural and base economy of the country.

### 3.5. Food source

Food scarcity is one of the consequences of climate change. Bamboo has the potential to improve food security in both human and livestock diets. The bamboo shoot is considered healthy food to consume in India and many Asian countries because of its high vitamin, mineral, fiber, protein, carbohydrates, and low-fat content. Many bamboo species, including *Bambusa balcooa*, have edible shoots high in protein and nutrients and are used in various cuisines [50]. The northeast region of India consumes bamboo shoots, which are prepared in a variety of dishes, either fresh or fermented traditionally, and some usually consumed bamboo foods include ushoi, mesu, soibum, eup, ekhung, and others [103]. They are used as an obesity controller because of their high fiber-rich source that regulates cholesterol levels and also enriches vitamin requirements of the body, such as vitamin 6, vitamin B3, vitamin B1, vitamin A and E [104]. A bamboo shoot develops a young culm composed of leathery sheaths compressed within internodes, which are harvested at 15–16 cm heights, and the internal portion can be washed and used as a food source after removing the fibrous sheath. *Phyllostachys edulis* and *Phyllostachys pubescens* are most commonly used to prepare various dishes [67]. A study examined *Dendrocalamus asper* as young shoot fiber culm flour used in food applications and discovered that it contains 67–79 g of fiber per 100 g. The remaining protein, carbohydrates, and lipids were also found to be above normal ranges, indicating that young bamboo culm flour can be used as a food source [105].

Another study was conducted in Kenya to use bamboo for frequent food storage to alleviate poverty and nutritional deficiencies. *Dendrocalamus giganteus, Bambusa vulgaris*, and *Yushania alpine* were studied for potential food sources. The results show high carbohydrate, protein, vitamin, and fiber content, as well as polyphenol, flavonoids, and antioxidant activity. This research establishes a foundation for using bamboo as a nutritious food source [106]. A similar study was carried out in India to assess the nutritional levels of bamboo. Several bamboo species were used, including *Bambusa balcooa, Bambusa bamboos, Bambusa vulgaris, Bambusa Wamin, Cephalostachyum pergracile, Dendrocalamus giganteus, Dendrocalamus strictus*, and *Gigantochloa manggong*, and their nutritional parameters, such as moisture, protein, carbohydrates, fiber, fats, vitamins, and so on, which were recorded as optimal for consumption as a food source [107].

Bamboo can be used in the food industry as sweeteners, sugars, prebiotic ingredients, and the pharmaceutical industry to prepare antioxidants and antibacterial products. The primary antioxidants found in bamboo shoots and leaves are vitamins C and E, phenols, and mineral elements like copper, iron, manganese, selenium, and zinc. Natural antioxidants are in great demand now because synthetic antioxidants found in food and medications may harm ones health [108]. The young bamboo culm flour had a low ash content (3 g 100 g^−1^), moisture content (10 g 100 g^−1^), protein, lipids, and, and the carb profile of the flour differed significantly in terms of sugar starch and total fiber. Fiber extraction was possible in all flour samples (> 60 g 100 g^−1^), but starch extraction was only possible in the *Bambusa vulgaris* and *Dendrocalamus asper* types (16 and 10 g 100 g^−1^, respectively) [105]. Bamboo shoots are a possible source of proteins for humans, with protein levels varying from 1.49 g 100 g^−1^ to 4.04 g 100 g^−1^ wet weight and 21.1 g 100 g^−1^ to 25.8 g 100 g^−1^ dry weight. The young bamboo shoots are considered gourmet delicacies in the Western world, where they are only available as imported canned products [109]. Bamboo shoots contain all eight or at least seven amino acids required by humans (Lys, Ser, Met, His, Ile, Leu, Phe). Compared to other vegetables, bamboo shoots have an unusually high free amino acid content, up to 4.03 g 100 g^−1^ in *Dendrocalamus hamiltonii* fresh shoots [110]. Cellulose and hemicellulose are potential biomass sources in biorefinery that are commonly used in bioprocess industries [111]. *Guadua amplexilofia* and *Dendrocalamus latiflorus* have high hemicellulose and cellulose fractions, providing increased productivity and decent biomass density while saving money on fertilizer and other requirements [112]. Various value-added products are obtained during the treatment and pre-treatment phases of the bioprocess, such as xylan, which is used as a prebiotic, and their multiple forms, such as xylose and xylitol, which are used as artificial sweetener residue for ethanol production.

## 4. Economy of bamboo plantation/biomass

Globally, bamboo is widely grown during plantation drives to encourage forest development as it has a short gestation time, extensive use for the building, pulpwood, flooring, panel items, and furniture [113]. Bamboo is a low-cost, accessible, and sustainable resource that outgrows most plants. The cultivation cost of bamboo in USD ha^−1^ in the initial year of cultivation and during total cultivation years was shown in Table 4. There are approximately 23 genera and 136 species of bamboo in India, with the North Eastern Region (NER) being especially rich in bamboo diversity, harboring more than half of the Indian bamboo species [50]. 35% of the total bamboo area and 66% of the growth stock of bamboo in India belongs to the NER [28,114]. Nearly 40% of India's overall bamboo cover is occupied by Odisha (7.39%), Arunachal Pradesh (9.36%), Maharashtra (9.63%), and Madhya Pradesh (13.04%) [28]. According to the 2011 census, agriculture and associated practices employ 54.6% of the Indian population but still only accounting for 17.4% of the countrys GDP [50]. Although India is the world’s second-largest bamboo producer, having a 19% of the world’s area in bamboo production, its bamboo exports still account for 6% of the global demand. India currently uses only 1/10th of its bamboo-growing capacity. Out of 136 species of bamboo, only around ten are presently being used commercially: *Bambusa affinis, Bambusa arundinacea, Bambusa balcooa, Bambusa tulda, Dendrocalamus asper, Dendrocalamus hamiltonii, dendrocalamus strictus, Ochlandra travancorica*, and *Oxytenanthera stocksii*. It has been estimated that 28 million metric tons (MMt) of bamboo demand in India could generate a massive 98,000 million man-days employment, especially in rural areas. Still, a case study from Purnia, Bihar, shows that only 15% of the people involved in bamboo-related industries earn more than 143.14 USD [61,115,116].

**Table 4.** Total bamboo cultivation cost in the initial year of cultivation and total cultivation year

Site	Cultivation cost (USD ha^−1^ y^−1^)	Cultivation cost (USD ha^−1^) (total cost)	Reference
Gujarat	624.57	--	[42]
Mahi ravines	594.44 (Initial year)	1420.35 (7 years)	[143]
Chambal Ravines	541.69 (Initial year)	1452.54 (7 years)	[143]
Yamuna ravines	572.30 (Initial year)	1568.96 (7 years)	[143]
ICAR-CARI, Jharkhand (10 m x 10 m plot)	50.88 (Initial year)	87.65 (5 years)	[113]
ICAR-CARI, Jharkhand (12 m x 10 m plot)	42.43 (Initial year)	79.22 (5 years)	[113]
Gujarat	1833.60	--	[42]
North Bihar	645.02	982.88 (2 years)	[144]

**Figure 3.**
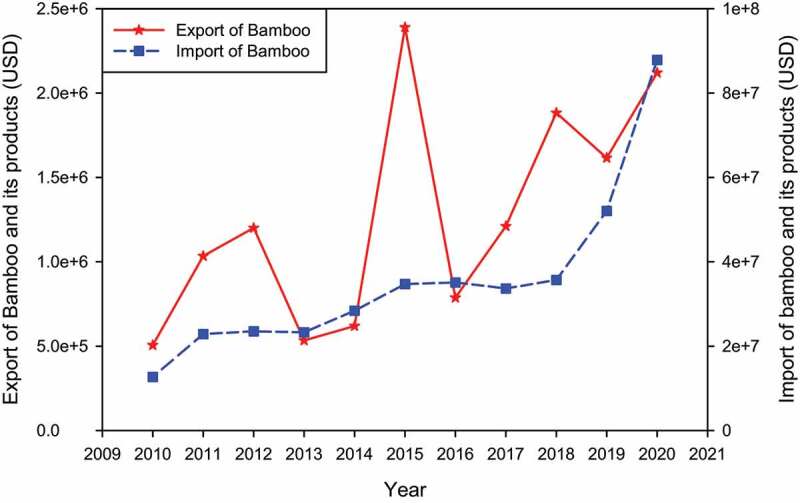
INBAR Bamboo trade database of years 2010–2020.

India was the sixth-largest economy in 2020, overtaking France, and is the fastest-growing trillion-dollar economy globally [29]. Approximate 20 million people are engaged in bamboo-related activities for their livelihood [115]. Every year the bamboo products produced in India are approximately 14.6 MMt with yearly productivity varying from 1–3 Mt y^−1^. Each year 9788 million USD and 7696 million USD are generated from the bamboo handicraft industry and construction, respectively. It was estimated that biomass cultivated in marginal degraded areas could generate approximately 12.83 MMt y^−1^ from the cottage industry products worth 16,616 million USD, potentially reducing bamboo import [42]. Bamboo provides two-thirds of India’s overall pulp supply for the paper industry [50]. Since bamboo is used in many countries for pulp and paper, it has a global exchange value of 26 million USD. India imports 1.2 million USD worth of pulp and paper-based products [28]. The foreign market value of incense stick/toothpick/chopsticks manufacturing industry articles is very high, with 1158 USD t^−1^, while pellet bulk prices in India are currently 232 USD t^−1^ [117]. The bamboo industry from *Bambusa Balcooa* can give employment to 8.66 million people indirectly through incense stick industry, production of bio-energy pellets from the waste of different bamboo associated industries, activated charcoal, etc., thus supporting government initiatives like Make in India and Skill India. Therefore, the bamboo industry encourages the growth of people from weaker economic sections and technology and helps in sustainable development, posing lesser to no threats to the environment [118]. The INBAR trade database of bamboo and its products for the years 2010–2020 were shown in Figure 3. INBAR stated that the overall annual production value of the global bamboo and rattan sector is around USD 60000 million. China is the world’s largest producer and exporter of bamboo, and its trade has increased in recent years. India and China account for 70% of Asia’s bamboo production and are the major bamboo-producing countries in the region. There are a total of 12 bamboo-based products traded in the global market. In 2018, India’s total recorded export of bamboo products was USD 1.96 million. With the growing trend in the global market for bamboo products, the country has a greater opportunity to grow in this segment.

**Figure 4.**
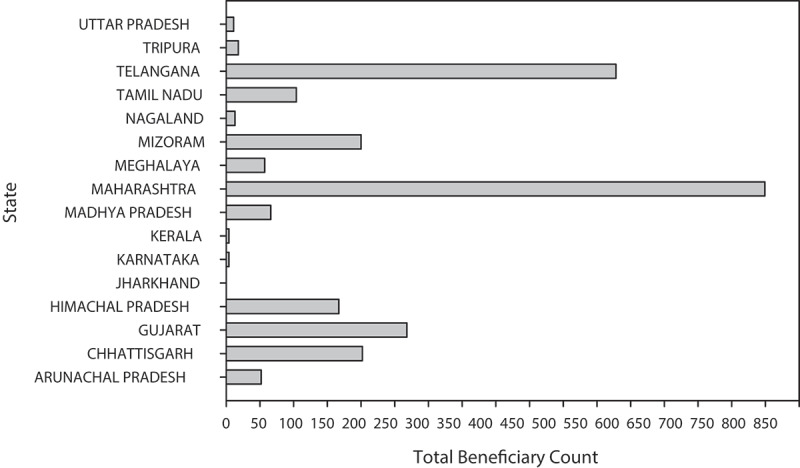
State-wise beneficiary count under national bamboo mission of financial years 2014–2021.

Unlike traditional brick walls, the bamboo reinforced prefabricated wall panels are 56% lightweight, 40% cheaper and cost-effective, and have good resilience, thus providing low-cost accommodation. It can potentially decrease the CO_2_ emissions generated during the fabrication of building materials like bricks, cement, steel, etc [119]. In 2019, India’s unemployment rate was 7.62%. In 2015, the bamboo industry in India generated a revenue of 3562.62 million USD [118]. Bamboo produces 432 million workdays a year in India, employing approximately 10 million people. The total bamboo cover in India can create about 129 million job opportunities. Telangana has the highest number of beneficiaries, as shown in Figure 4, which depicts the state-by-state beneficiary count from 2014–2022. Bamboo entrepreneurship in rural as well as urban areas, create various income opportunities like the manufacture of agarbatti, pencils, matchbox, chopsticks, lathis, fishing rods, housing materials, building materials, doors, windows, interiors design, furniture, bridges, ladder, laminated window frames and flooring tiles, artificial boards, powdered and granular activated charcoal, fabrics like sweaters, mats, blankets, towels, sanitary products, food fodders, and fertilizers. Small-scale industries of bamboo produce 41.11 million USD in revenue in India [118].

The energy sector is critical for a country’s economic growth. Rising global energy demand has resulted in a fossil fuel shortage. Therefore, alternative energy sources must be investigated. Renewable energy sources account for 19% of all energy sources globally, with biomass accounting for 14% [117]. The waste generated from the bamboo cottage industry can be converted into 13,987 million USD worth of bioenergy pellets, replacing 63.18 MMt quantity of coal. Waste produced from the handicraft industry using bamboo may also be used for bioenergy ventures, resulting in 20–25 t of CO_2_ acre^−1^ y^−1^ sequestration [120]. There are around 146 million hectares of marginal land in India. Farmers selling raw bamboo grown on the wasteland can earn up to 800 USD per hectare annually. Bamboo production can generate roughly ten certified emission reductions each hectare per year, sold as carbon credits. Furthermore, underemployed farmers working in the bamboo handicraft industry can earn up to 2700 USD y^−1^ as per current exchange rates, which is considerably more than the current average earnings of farmers that is 1750 USD y^−1^ hence improving the average earnings of the farmers. Bamboo plantation needs lesser amounts of chemical amendments, making it cost-effective and eco-friendly [121].

*Dendrocalamus strictus*, the most widespread species in India, can be a potential alternative for agroforestry systems in semi-arid tropical regions, improving yield and commercial returns [61]. The utilization of bamboo can be economically unappealing due to inadequate procedures, excessive logistic expenses, excessive waste, poor management, etc. Therefore, an integrated approach involving multiple industries is applied for better and efficient bamboo biomass utilization to reduce marketing and logistics costs. Cultivation of bamboo on marginal degraded lands is the most feasible, cost-effective, and environmentally sustainable option to promote GHGs and CO_2_ mitigation. Patel et al. [42] compared the production and profit earned after the expenditure of 1 USD on the cultivation of bamboo, sorghum, and pearl millets and found that the bamboo plants showed the highest profit of 4.83 USD, biomass production of 55.69 kg, and net energy balance of 0.96 GJ and lowest GHG emission potential with 1.65 Kg CO_2_eq emissions. Thus, indicating that bamboo cultivation can contribute to the Indian economy if utilized efficiently along with proper policies formulated for enhancing the cause. In Aravalli district, North Gujarat, India, high-biomass bamboo with a yield of 20–25 t acre^−1^ has been grown on 120 acres as a captive farming and agroforestry system to monetize marginal lands [117]. The most widely found bamboo species in India is *Dendrocalamus strictus*, comprising around 53% of the total area covered by all bamboo species found in India. As compared to the timber wood species like eucalyptus, popular, etc., *Dendrocalamus strictus* has lesser cultivation and upkeep costs, yielding culms commercially after 4–5 years of plantation, giving harvest annually until flowering occurs. *Dendrocalamus strictus* used in an agroforestry system with *Sesamum indicum* (10 × 10) had the highest benefit-cost ratio of 2.83 indicating that bamboo could be a better alternative as compared to conventional agroforestry in semi-arid tropics region [113].

The economic analysis of the bamboo plantation (*Dendrocalamus strictus*) on the degraded Yamuna ravines suggested that each party involved in the area could earn 457.37–654.37 USD ha^−1^ after the 7th year of the cultivation with a cost-benefit ratio of 1.89 in the region along with benefitting the environment by rejuvenating the degraded soil [122]. Patel et al. [120] observed that 1,112 plants per hectare of *Bambusa balcooa* planted by abellon clean energy in Gujarat were the optimum plantation density after the field trials at four plantation densities per hectare which produced 57.70 Mt ha^−1^ biomass yield having 32–39 USD Mt^−1^ production cost. It is reported that the utilization of 5% of the degraded land by cultivating *Bambusa Balcooa* with 50% biomass yield can generate 67.51 MMt y^−1^ of bamboo biomass. So by encouraging bamboo plantation across the country, many social, economic, and environmental concerns can be solved including the creation of job opportunities in farming, self-employed business, rural poverty, soil loss, deforestation, urban development, inefficient resource usage, bioenergy, and GHG emission mitigation by utilizing pellets to replace LPG gas, lowering imports of fossil fuel and efficient manufacturing and utilization of bamboo products and raw material, and thus maintaining the inflow of money within the country, resulting in increasing GDP [61,123].

## 5. Opportunities, challenges and prospects

Due to the immense growth in population, urbanization, and modernization, the demand for food and energy has increased to meet the concern of the scenario. Bamboo has emerged as a potential biomass feedstock species as well as an alternate bioenergy source [124]. Because of their fast growth and ability to grow in degraded lands, they can be used to restore wasteland or degraded land, and have a long root system that can prevent soil erosion and landslides in hilly regions. Bamboo has emerged as a key player in bioenergy production due to its ease of availability, social and economic feasibility for the prospects [58]. Although the socioeconomic and ecological benefits of bamboo are phenomenal, the need of the hour is to use it as a wholesome product like for land degradation savoir, as food material, medicinal use, and to contribute to bioenergy production by using it in other ways at the same time. The widespread use of bamboo could contribute to food security while also preserving biodiversity.

Bamboo should be promoted as a potential feedstock due to its high biomass production capacity and as a commercial source of the bioprocess industry by directly contributing to the production of next-generation fuels such as bioethanol [125]. The commercialization of these bamboo lignocellulosic biorefineries in the near future has the potential to revolutionise India’s entire energy sector. Such plantations would contribute to the overall development of rural livelihoods while also contributing to the nation’s economy in environmentally friendly ways. More research is needed to effectively produce biofuel from lignocellulosic biomass pre-treatment via the saccharification process [94]. Sugars are released during the pre-treatment process, which aids in the bioethanol production process from bamboo biomass. For the production of biofuel from bamboo in biorefinery systems to be feasible, advances in agronomy, genetics, and bioconversion are required.

In India, climate change mitigation has been gaining attention via bamboo plantations. Bamboo can generate carbon credits that can be sold globally because of its high carbon sequestration rates. As they can regulate carbon sequestration level, and provide fodder and meet the timber, food, and basic requirements of the local rural peoples, it aids in agroforestry by converting degraded land into bamboo forests [50]. Bamboo plantation can also provide employment and promote entrepreneurship, as well as contribute to a significant increase in the value of bamboo products, allowing industries to earn profits and contribute to the national economy, among other things. These are the areas where India is progressing through the use of bamboo at various stages. Even though, it appears that the key bottleneck glitches prevailing in bamboo plantation are associated with dearth of awareness of bamboo important as well as a dearth of adequate consideration to its commercialization. Thus, the governmental bodies and national drives can assist to increase the socio-economic and environmental awareness related to bamboo and its products. Therefore, to make bamboo as a potential crop for upfitting the socio-economic statues of the individuals as well as the nation, a central and state level-based policies/laws need to be developed and strictly implemented.

The bamboo associated policies needs to go hand in hand with the consolidation of Ministries that accomplish these policies into one authority that will be authorized to investigate and reform the policies as needed and to support policies with the precise incentive and institutional structure to develop bamboo sector. Bamboo industry sectors need to be incentivized to speed up the expansion of bamboo industry. This is exclusively imperative owing to the enormous possibility it holding in improving the farmers income. To provide motivation to the application of bamboo in construction, environmentally friendly government building should be constructed. Current utilization of bamboo and its products especially in the building purposes is not inspiring. It is observed that some of the bamboo-based industries that running currently are not operating at its full capacity, which need to be upgraded. Furthermore, a policy-based decision needs to be adopted to warrant that a certain percentage of all government buildings, such as offices, residences, schools, school furniture quarters barracks of paramilitary forces, etc utilize bamboo and bamboo-based products to boost this environmentally friendly industry.

Majorities of research work on bamboos has been covered up the aspects of its commercial farming and the applications, such as approaches to maximizing its biomass yields and on delivering economic evaluations of cropping in diverse contexts. So far, only a few investigations have been performed, which evaluated the invasive behaviour of bamboos, in spite of their characteristics to a group having high ‘invasive’ species [126,127]. Bamboo cropping have been revealed as a significant ecological driver in various regions of the globe, however, its invasive nature is impeding the natural growth of the secondary forests leads to losses to the biodiversity and production yield, which ultimately leads to economic losses of cultivars. However, up to now only few studies have verified the effect of bamboo plantation on other natural plant species [127,128]. Although, evidence-based facts are available recently to support discussions related to how bamboo invasion adversely impacting the native plant species. However, detail studies on economical losses posed by bamboo invasion are still lacking, which need to be emphasized in future long-term field-oriented research studies.

## 6. Conclusion

The socioeconomic and environmental benefits of bamboo in India, along with the production of bioenergy, are investigated in this paper. Bamboo has many potentials to be used as a feedstock to produce bioenergy in India if it is harvested, managed, and planned properly. Bamboo is widely available, well-known among locals, fast-growing, versatile, quickly storing and sequestering carbon, growing in degraded lands, and has excellent fuel properties for conventional bioenergy production. Incorporating multifunctional perennial bamboo crops into energy sources could significantly contribute to achieving renewable energy goals and land rejuvenation objectives by reducing the exorbitant prices associated with meeting the restoration goals. Additionally, this review urges researchers to explore the ways to produce and process bioenergy in the country and explore whether these approaches will reduce GHG emissions. Furthermore, research on bamboo restoration economics and incentives, including profitability and return on capital to landowners, are required.

## CRediT authorship contribution statement

**Rashmi Rathour**: Conceptualization, writing-original draft, editing, validation, and visualization. **Hemant Kumar**: Writing-original draft, editing, validation, and visualization. **Komal Prasad**: Writing-original draft, validation, and editing. **Prathmesh Anerao**: Writing-original draft, validation, and editing. **Manish Kumar**: Conceptualization, writing-original draft, validation, editing, and visualization. **Atya Kapley**: Writing-original draft, validation, and editing. **Ashok Pandey**: Conceptualization, writing-original draft, validation, and editing. **Mukesh Kumar Awasthi**: Conceptualization, writing-original draft, editing, validation, visualization, and supervision. **Lal Singh**: Conceptualization, writing-original draft, editing, validation, visualization, supervision, fund acquisition, and project administration.
